# Cardiac arrhythmias during or after epileptic seizures

**DOI:** 10.1136/jnnp-2015-310559

**Published:** 2015-06-02

**Authors:** Marije van der Lende, Rainer Surges, Josemir W Sander, Roland D Thijs

**Affiliations:** 1 Stichting Epilepsie Instellingen Nederland (SEIN), Heemstede, The Netherlands; 2 Department of Neurology, Leiden University Medical Center (LUMC), Leiden, The Netherlands; 3 Department of Epileptology, University of Bonn Medical Center, Bonn, Germany; 4 Department of Clinical & Experimental Epilepsy, NIHR University College London Hospitals Biomedical Research Centre, UCL Institute of Neurology, London, UK; 5 Epilepsy Society, Chalfont St Peter, UK

**Keywords:** EPILEPSY, SUDDEN DEATH, CARDIOLOGY

## Abstract

Seizure-related cardiac arrhythmias are frequently reported and have been implicated as potential pathomechanisms of Sudden Unexpected Death in Epilepsy (SUDEP). We attempted to identify clinical profiles associated with various (post)ictal cardiac arrhythmias. We conducted a systematic search from the first date available to July 2013 on the combination of two terms: ‘cardiac arrhythmias’ and ‘epilepsy’. The databases searched were PubMed, Embase (OVID version), Web of Science and COCHRANE Library. We attempted to identify all case reports and case series. We identified seven distinct patterns of (post)ictal cardiac arrhythmias: ictal asystole (103 cases), postictal asystole (13 cases), ictal bradycardia (25 cases), ictal atrioventricular (AV)-conduction block (11 cases), postictal AV-conduction block (2 cases), (post)ictal atrial flutter/atrial fibrillation (14 cases) and postictal ventricular fibrillation (3 cases). Ictal asystole had a mean prevalence of 0.318% (95% CI 0.316% to 0.320%) in people with refractory epilepsy who underwent video-EEG monitoring. Ictal asystole, bradycardia and AV-conduction block were self-limiting in all but one of the cases and seen during focal dyscognitive seizures. Seizure onset was mostly temporal (91%) without consistent lateralisation. Postictal arrhythmias were mostly found following convulsive seizures and often associated with (near) SUDEP. The contrasting clinical profiles of ictal and postictal arrhythmias suggest different pathomechanisms. Postictal rather than ictal arrhythmias seem of greater importance to the pathophysiology of SUDEP.

## Introduction

The occurrence of asystole during the course of an epileptic seizure was described well over 100 years ago: “He uttered a cry and was seen to be rubbing his hands together. His pulse was immediately examined for but was not palpable.”^1^ Since then, various ictal cardiac arrhythmias have been reported, and it has been acknowledged that seizures can influence cardiovascular control.

Sinus tachycardia is the most common cardiac consequence of epileptic seizures and may occur in up to 80% of seizures.^2^ It may be associated with palpitations, but not with clinical signs such as syncope. Of all clinically relevant ictal arrhythmias, ictal asystole has gained much attention as it may cause syncope and subsequent falls, fractures and traffic accidents.^3^ Ictal asystole and rarer ictal arrhythmias have also been suggested as a potential pathomechanism for Sudden Unexpected Death in Epilepsy (SUDEP).

As most information derives from single case reports and case series, our view on ictal arrhythmias still remains fragmented.

We systematically reviewed the literature to identify the full spectrum of clinically relevant (post)ictal cardiac arrhythmias attempting to unveil clinical profiles associated with each arrhythmia.

## Methods

We performed a systematic review from the first date available to July 2013 and searched PubMed, EMBASE (OVID version), Web of Science and the COCHRANE Library. We used subject queries taking into account the terminological differences between these databases. Queries consisted of the combination of two terms: ‘cardiac arrhythmias’ and ‘epilepsy’. Various synonyms and related terms for all subjects were used (for exact search strategy, see online supplementary appendix A).

One author (MvdL) screened all titles and abstracts for case series and case reports on ictal cardiac arrhythmias. Articles relating to cardiac arrhythmias mistaken for epileptic seizures, medication-induced arrhythmias, animal studies, interictal cardiac arrhythmias and sinus tachycardia were excluded.

Full texts of all remaining articles were screened. We selected all those with data on individual cases of the following arrhythmias: asystole, bradycardia, atrioventricular conduction block, postictal atrioventricular conduction block, atrial fibrillation (AF)/flutter, ventricular tachycardia/fibrillation and pre-excitation syndromes including Wolff-Parkinson-White. For each individual case, we recorded whether the onset was in the preictal, ictal or postictal phase. Asystole was defined as an R-R interval of >3 s. Bradycardia was defined as a heart rate under the first centile of a normal heart rate frequency in bpm.^4^

Reviews were screened to find additional cases. We also reviewed articles from our personal archives. Only cases with simultaneous video-EEG (vEEG) recordings were included, apart from arrhythmias with fewer than five identified case reports with vEEG. For each case, the following variables were collected: age, gender, type of epilepsy, duration of epilepsy, seizure frequency, number and type of antiepileptic drugs taken, handedness, brain MRI, seizure type associated with cardiac arrhythmia, duration of arrhythmia, time between seizure onset and arrhythmia, localisation of seizure onset, cardiac history, pacemaker implantation. Data from individual cases were collected into databases for each arrhythmia. To determine the prevalence of ictal asystole, we combined individual data from similar studies.

## Results

One thousand one hundred and sixty-seven articles were identified, and after titles and abstracts were reviewed, we excluded 989. After 178 full text articles were reviewed, 65 reporting 162 cases with (post)ictal arrhythmias were included (see online supplementary appendix B). No preictal cardiac arrhythmias were identified.

### Bradycardia

#### Asystole

After exclusion of 13 cases without vEEG data, 126 cases of asystole were included (14 case series; 43 case reports), 103 with ictal and 13 with postictal onset.

##### Ictal asystole

Prevalence data were reported in seven case series. Asystole was defined as an R-R interval of >3 s in two case series and an R-R interval of >4 s in one case series. The remaining four case series did not provide a definition of asystole. The mean prevalence of ictal asystole in all people admitted for a vEEG recording (including those without epilepsy) was 0.177% (95% CI 0.177% to 0.178%).^5^ ^6^ The mean prevalence of ictal asystole in all people with refractory focal epilepsy admitted for a vEEG recording was 0.318% (95% CI 0.316% to 0.320%).^7–11^

Ictal asystole was only reported in people with focal epilepsy (table 1). Most of the ictal asystoles occurred during the course of a focal dyscognitive seizure (formally known as a complex partial seizure), on average starting 30 s after seizure onset. The mean duration of ictal asystole was 20 s (range 3–96). The seizure onset zone was reported in 78% of the cases and was temporal in 90% without consistent lateralisation.

**Table 1:** (Post)ictal asystole

	Ictal asystole n=103	Reported in n cases	Postictal asystole n=13	Reported in n cases
Age (years)	44 (16.3)	101	34 (11.7)	13
Gender	51% male	101	46% male	13
Type of epilepsy	100% focal	89	100% focal	12
Epilepsy duration (years)	15 (5–30)	60	8 (2–21)	11
Seizure frequency (per month)	4 (1–10)	25		
AED	No AED 13%	84		
	Monotherapy 27%			
	Polytherapy 60%			
Normal MRI	50%	64	17%	6
Right handed	92%	36		
Seizure duration prior to asystole (s)	24 (13–35)	47	187 (71–276)	8
Time between seizure offset and start of asystole (s)			90 (20–158)	10
Seizure type at onset of asystole	99% FDS	96	85% fbCS	13
	1% FAS	1	15% FDS	
Evolving to bilateral convulsive seizure after onset of asystole	7%	90	Not applicable	
Duration asystole (s)	19 (10–26)	96	24 (7–60)	6
EEG seizure onset (n)	LT/LFT 37 (35/2)	80	LT/LFT 2 (2/0)	10
	RT/RFT 25 (23/2)		RT/RFT 6 (5/1)	
	BT 10		BF 1	
	LH 3		Ri par 1	
	Non-lat 3			
	RH 1			
	RO 1			
PGES before asystole	Not applicable		70%	10
Apnoea before asystole			100%	8
Pacemaker implanted	88%	50	50%	4

Results are presented as percentiles, mean (SD) or median (25th–75th centile).

AED, antiepileptic drugs; BF, bifrontal; BT, bitemporal; FAS, focal autonomic seizure; fbCS, focal seizure evolving to bilateral convulsive seizure; FDS, focal dyscognitive seizure; LFT, left frontotemporal; LH, left hemisphere; LT, left temporal; non-lat, non-laterising; PGES, postictal generalised EEG suppression; RFT, right frontotemporal; RH, right hemisphere; ri par, right parietal; RO, right occipital; RT, right temporal.

All ictal asystoles were self-limiting, except in one subject where resuscitation was started after 44 s of cardiac arrest. This event was labelled as near-SUDEP.

##### Postictal asystole

Most of the postictal asystoles were seen after a focal seizure evolving to a bilateral convulsive seizure and had a mean duration of 30 s (table 1). They were preceded by postictal generalised EEG suppression (PGES). Seven of 13 people died of (probable) SUDEP.

#### Ictal bradycardia

Twenty-five vEEG cases of ictal bradycardia without asystole were identified. Characteristics of ictal bradycardia cases were similar to those with ictal asystole. Ictal bradycardia was only reported in people with focal epilepsy during focal dyscognitive seizures. Seizure onset was predominantly temporal (table 2).

**Table 2:** Ictal bradycardia

	Ictal bradycardia n=25	Reported in n cases
Age (years)	48 (22.5)	20
Gender	55% male	20
Type of epilepsy	100% focal	21
Epilepsy duration (years)	5 (0–9)	10
AED	No AED 22%	9
	Monotherapy 44%	
	Polytherapy 33%	
Normal MRI	38%	13
Right handed	60%	5
Seizure duration prior to bradycardia (sec)	25 (11–39)	9
Seizure type at onset of bradycardia	100% FDS	8
EEG seizure onset (n)	LT/LFT 11 (8/3)	21
	RT/RFT 8 (7/1)	
	T 1	
	L par occ 1	
Pacemaker implantation	3 (37%)	8

Results are presented as percentiles, mean (SD) or median (25th–75th percentile).

AED, antiepileptic drugs; FDS, focal dyscognitive seizure; L par occ, left parieto-occipital; LFT, left frontotemporal; LT, left temporal; sGTCS, secondary generalised tonic clonic seizure; T, temporal.

#### Ictal atrioventricular-conduction block

We found 11 cases of ictal atrioventricular (AV)-conduction block: 9 complete AV blocks and 2 second-degree AV blocks. In five cases, complete ictal AV block was followed by a cardiac standstill; these cases were also included in the ictal asystole section. A pre-existent conduction block (left or right bundle branch block) was reported in 2 of 11 cases. All had focal epilepsy. All ictal AV blocks occurred during non-convulsive seizures. Seizure onset was never lateralised primarily in the right hemisphere (table 3).

**Table 3:** (Post)ictal AV block

	Ictal AV conduction block (n=11)	Reported in n cases	Postictal AV conduction block (n=2)	Reported in n cases
Age (years)	49 (12)	11	30 and 56	2
Gender	20% male	10	1 male, 1 female	2
Type of epilepsy	100% focal	10	focal	1
Epilepsy duration (years)	23 (11–31)	5	39	1
Seizure type	90% FDS	10	100% fbCS	2
	10% FAS			
EEG seizure onset (n)	LT/LFT 8 (7/1)	11	RT	1
	BT=2			
	Left insula=1			
Pacemaker implanted	100%	5	100%	1

Results are presented as percentiles, mean (SD) or median (25th–75th percentile).

AV, atrioventricular; BT, bitemporal; FAS, focal autonomic seizure; fbCS, focal seizure evolving to bilateral convulsive seizure; FDS, focal dyscognitive seizure; LFT, left frontotemporal; LT, left temporal; RT, right temporal.

#### Postictal AV-conduction block

Two cases of postictal AV blocks were found; both were preceded by a focal seizure evolving to a bilateral convulsive seizure.

### Tachycardia

No cases with ictal AV nodal tachycardia, AV re-entry tachycardia or pre-excitation syndromes such as Wolff-Parkinson-White were identified.

#### Atrial flutter/atrial fibrillation

Fourteen cases of (post)ictal AF (n=13) or atrial flutter (n=1) were found. Only three participants had an ictal vEEG recording: one ictal AF during a focal dyscognitive seizure and two cases of postictal AF after convulsive seizures; two of these cases later died of definite SUDEP.

The remaining 11 cases without vEEG had AF after a possible convulsive seizure, usually persisting for several hours. Owing to the lack of ictal proof, these cases are discussed separately (table 4).

**Table 4:** (Post)ictal atrial fibrillation

	(Post)ictal AF with vEEG (n=3)	Reported in n cases	(Post)ictal AF without vEEG (n=10)	Reported in n cases
Age (years)	22, 34	2	37 (16)	10
Gender		0	90% male	10
Type of epilepsy	2 focal epilepsy, 1 GGE	3	5 focal epilepsy, 3 GGE	8
Epilepsy duration (years)	6, 34	2	7 (0–25) SD 10.3	7
Seizure frequency	1/year, 1/week	2	3/year, 3/week	2
Seizure type	1 GTCS, 1 fbCS, 1 FDS	3	50% fbCS, 50% GTCS	10
Start of AF in postictal phase	2	3		
Duration of AF	10 s, 55 s, >110 s	3	1.5–25 h	9
Normal MRI		0	57%	7
Cardiac history	0%	2	14%	7
EEG seizure onset	Non loc, LT, Gen	3		0

Results are presented as percentiles, mean (SD) or median (25th–75th centile).

AF, atrial fibrillation; fbCS, focal seizure evolving to bilateral convulsive seizure; FDS, focal dyscognitive seizure; Gen, generalised; GGE, genetic generalised epilepsy; GTCS, generalised tonic clonic seizure; LT, left temporal; non loc, non-localising; vEEG, vedio-EEG.

#### Ventricular fibrillation

Three cases of postictal VF were identified, one without vEEG. In all three cases, VF was preceded by a convulsive seizure and cardiopulmonary resuscitation (CPR) was initiated: two were classified as near-SUDEP and one as definite SUDEP.

One individual was known to have a first-degree AV block, but none had major ventricular tachycardia/ventricular fibrillation (VT/VF) risk factors.

## Discussion

**Figure 1:**
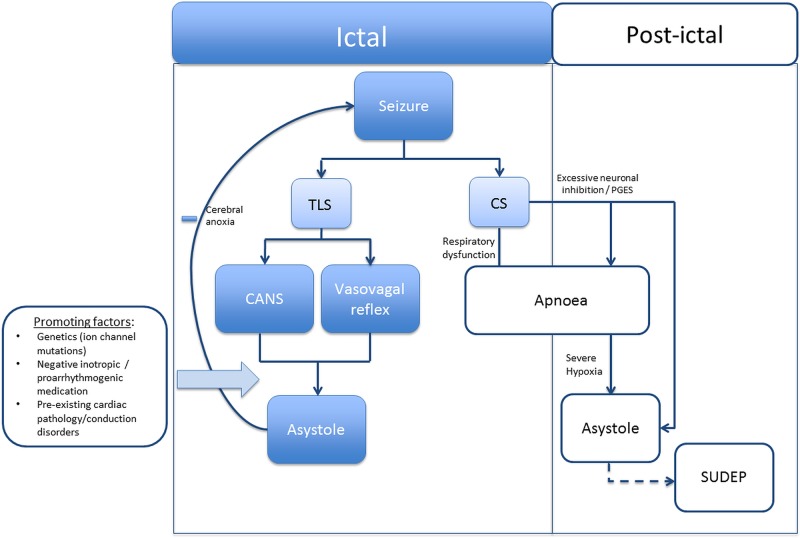
Schematic overview of the mechanisms for seizure-related asystole. Ictal asystole is strongly associated with temporal lobe seizures. It could be a direct consequence of epileptic activity stimulating the central autonomic network or an indirect effect of the seizure (eg, catecholamine release) evoking a vasovagal reflex. Ictal asystole is self-limiting, as cerebral anoxia caused by the asystole ceases the seizure. In contrast, postictal asystole is associated with convulsive seizures and could be fatal. Postictal apnoea is often preceded by apnoea and/or PGES. Prolonged apnoea eventually causes arousal as well as bradycardia and asystole. Postictal coma may, however, block the arousal effect and thus the resumption of ventilation, explaining why postictal asystole may lead to SUDEP. SUDEP, sudden unexpected death in epilepsy; TLS, temporal lobe seizure, CS, convulsive seizure, CANS, central autonomic nervous system; PGES, postictal generalised EEG suppression.

Seven distinct (post)ictal arrhythmia patterns were identified. Ictal asystole was the most frequently reported pattern. Ictal asystole, ictal bradycardia and ictal AV block predominantly occurred during focal dyscognitive seizures in people with temporal lobe epilepsy. No deaths were reported suggesting that ictal arrhythmias are self-limiting. In contrast, postictal arrhythmias including asystole, AV block and the less prevalent AF and VF usually occurred after a convulsive seizure and were frequently associated with (near-)SUDEP. The difference in timing, associated seizure types and mortality risk suggests that seizures may trigger cardiac arrhythmias in various ways. Postictal arrhythmias, rather than ictal arrhythmias, seem of greater importance to the pathophysiology of SUDEP (figure 1).

### Limitations

Publication biases are inevitable and may affect results. Reliable estimates of prevalence could only be made for ictal asystole, among those with refractory epilepsy, yielding an overall prevalence of 0.32%. Variable definitions were used (R-R interval of >3 s, >4 s, no definition at all); thus, the prevalence number might be underestimated. For the other arrhythmias, no estimations are available, but the few case reports and the lack of case series suggest that they are rare. Selection bias may also have been at play as evaluation for epilepsy surgery is the most common indication for vEEG registration; thus, people with temporal lobe epilepsy may have been over-represented. Only one case series was reported on epilepsy types: almost twice as many people with temporal lobe epilepsy were monitored with vEEG compared to those with extra temporal lobe or generalised epilepsy.^6^ This may have resulted in an overestimation of the association of temporal lobe epilepsy in those with ictal and postictal asystole.

Diagnostic validity is another potential limitation as we included cases of AF (n=11) and VF (n=1) without vEEG. In these cases, we were unable to verify the seizure and determine the exact time of onset of the arrhythmia. Therefore, we reported those cases separately. Overall, no major differences were seen between the two groups, particularly with respect to the relationship with seizure type and timing of the arrhythmia.

Muscle artefacts, particularly those during convulsive seizures, may have obscured the detection of ictal arrhythmias with a single lead ECG channel. This limitation may apply to all arrhythmias except for those causing (pre)syncope, as this will become apparent by a sudden diffuse slowing and flattening of the EEG.^12^ Nevertheless, it should be noted that the prevalence of ictal arrhythmias without syncope might have been underestimated.

Many case reports did not report on cardiac history, use of cardiovascular drugs, withdrawal of antiepileptic drugs, baseline ECG or ECG pattern preceding the arrhythmia. For example, two case series reported AV conduction time prior to asystole and found that 5 of 16 cases had complete AV block.^7^ ^13^ As other case series did not provide these data, ictal AV block may be more or less common than our data suggest. We were also unable to assess the influence of cardiac history on ictal arrhythmias, or the possible arrhythmogenic effects of medication. We would strongly recommend that future case series provide details on cardiac history, prior medication use, baseline ECG and ECG pattern to allow for such analyses.

### Ictal asystole, bradycardia and AV block

We found a point prevalence of people with ictal asystole of 0.32%. In contrast, two small prospective studies (both n=19) with long-term implantable heart rhythm monitors up to 2 years reported a much higher prevalence of 5% and 21%.^14^ ^15^ These contrasting figures suggest that ictal asystole does not occur during every seizure and may go unnoticed during short-term monitoring.

Ictal asystole, ictal bradycardia and ictal AV block coincided with a focal dyscognitive seizure and were predominantly seen in temporal lobe epilepsy. These three arrhythmias not only shared a similar clinical profile, but could also overlap. Both ictal bradycardia and ictal AV block may evolve into asystole.

It has been suggested that a seizure onset in the left hemisphere results in bradycardia and that a right-sided onset results in tachycardia.^2^ We did not, however, find a consistent lateralisation in the large group of ictal asystole and ictal bradycardia cases. In the small group of ictal AV block cases, there was a tendency for a left-sided focus. We cannot exclude the possibility that seizure lateralisation is relevant only for ictal AV block, as it is known that the left vagal nerve predominantly innervates the atrioventricular node and the right vagal nerve innervates the sino-atrial node. In view of the possible overlap between ictal asystole and ictal AV block,^7^ ^13^ and the fact that most ictal asystole studies did not take this overlap into account, this would need larger studies for definite confirmation.

Ictal asystole could be a direct consequence of epileptic activity stimulating the central autonomic network.^2^ ^16^ Focal stimulation of parts of the limbic system, such as the cingulate gyrus, amygdala, and insular and orbitofrontal cortex, may provoke asystole.^17–19^

Ictal asystole may be promoted by the use of drugs (eg, effecting AV conduction or sinoatrial node activity) or genetic conditions affecting cardiac conduction (ion channel mutations).

Prospective long-term studies suggested that ictal asystole may be incidental (on average, 1 of 13 seizures within the same patient with ictal asystole).^14^ ^15^ The variable expression between seizures could argue that ictal asystole is caused by an *indirect* effect of the seizure.

Ictal asystole may parallel centrally mediated cardioinhibition seen in vasovagal syncope. As in emotionally induced vasovagal syncope, seizure-induced fear and catecholamine release may coincide and culminate in cardioinhibition and vasodilation.^20^ ^21^

Supporting this view, heart rate patterns preceding asystole were similar between subjects with ictal asystole and those with vasovagal syncope: heart rate increases markedly, followed by a progressive bradycardia, leading to asystole.^22^ Vasovagal syncope is a self-limiting condition with an excellent long-term prognosis.^23^ Prolonged cerebral hypoperfusion is thought to shut down the initial central trigger, thereby explaining its benign course.^12^ Following this analogy, cerebral anoxia-ischaemia in ictal asystole could be a potential mechanism of seizure self-termination as well. Accordingly, the total seizure duration was found to be shorter for seizures with ictal asystole compared to those without.^24^

The most extreme case of self-limiting ictal asystole that we found lasted for 96 s.^25^ In the single ictal asystole case with near-SUDEP,^7^ successful resuscitation was started after 44 s of cardiac arrest. Whether an asystolic event is labelled as near-SUDEP or as a self-limiting ictal asystole will thus critically depend on the action of the observing medical personnel: immediate resuscitation will increase the number of ‘near-SUDEP’ cases. Therefore, as long as no fatal case has been reported, ictal asystole should not be considered a SUDEP pathomechanism.

### Postictal asystole

We found that postictal asystole is associated with convulsive rather than focal dyscognitive temporal lobe seizures and is frequently associated with SUDEP. Most of the postictal asystoles were preceded by PGES and apnoea. In the MORTEMUS study,^26^ vEEG recordings were used to estimate the presence of respiratory movements; all postictal asystoles were most likely preceded by apnoea.

Prolonged apnoea activates the carotid chemoreceptors, causing arousal and eventually vagally mediated bradycardia or even cardiac arrest.^27^ In the context of postictal coma, the arousal effect may be blocked and thus not result in resumption of normal ventilation, thus explaining why postictal asystole can be fatal. PGES has been linked to postictal coma.^28^ ^29^ The exact mechanism underlying PGES and subsequent cardiorespiratory cessation remains unexplained, but may result from excessive brainstem inhibition.^26^

### Postictal AF and VF

Postictal AF and VF were detected in the context of convulsive seizures and, in contrast with ictal asystole and ictal bradycardia, AF was usually present for several hours. Postictal VF is always classified as (near-)SUDEP.

Convulsive seizures trigger the sympathetic nervous system as reflected by a peak in catecholamine and electrodermal activity.^30^ ^31^ Increased sympathetic activity has also been implicated as a trigger for AF and VF.^32^

People with epilepsy were found to have a threefold increased risk of VT/VF compared with the general population.^33^ Most cases of VT/VF in epilepsy were, however, not seizure-related and were probably related to cardiovascular comorbidities. Nevertheless, in a subset of cases, seizure-induced VF may have played a role.^34^

As well as a rise in catecholamines,^35^ various other factors may contribute to postictal VF, including a higher prevalence of ECG markers for sudden cardiac arrest;^36^ peri-ictal QTc prolongation,^35^ ^37^ ST changes^38^ and increased troponin levels.^38^ ^39^ Possibly all these factors converge over time, thus explaining the occurrence in the postictal phase.^40^

### Clinical implications

In view of ictal asystole's self-limiting course, a reasonable approach is to optimise treatment with antiepileptic drugs, to consider epilepsy surgery, and to withdraw negative inotropic or proarrhythmogenic drugs.^11^ If this fails and there is documented recurrence of asystolic episodes, cardiac pacemaker implantation should be considered. Observational studies suggest that pacemakers can reduce falls and injuries due to seizure-induced syncope.^3^ ^11^

Postictal arrhythmias may be a marker of an increased SUDEP risk. No studies have yet addressed the management of these cases. In the absence of such evidence, we recommend optimisation of seizure control and a critical review of the clinical context (eg, drug use, ECG markers) in order to identify other modifiable risk factors.

## Supplementary Material

Web appendix 1

Web appendix 2
