# Supplementary material for: Cardiac arrhythmias during or after epileptic seizures
Source: J Neurol Neurosurg Psychiatry. 2015 Jun 2;87(1):69–74. doi: 10.1136/jnnp-2015-310559 (PMC4717443; doi:10.1136/jnnp-2015-310559)
Supplement: Web appendix 1 [file jnnp-2015-310559-s1.pdf]

Appendix A Search strategy

Total 2-7-2013: 1.158 references:

- PubMed: 800 [RefID 1-800]
- Embase (no meeting abstracts): 807, 329 unique [RefID 801-1239]
- Web of Science (no meeting abstracts): 207, 9 unique [RefID 1240-1259]
- COCHRANE: 23 RCTs, 20 unique [RefID 1260-1280]
- * missing RefIDs are manually deleted references

Databases:

PubMed
http://www.ncbi.nlm.nih.gov/entrez/query.fcgi?otool=leiden

("Arrhythmias, Cardiac"[majr] OR "Death, Sudden, Cardiac"[Majr] **OR "Heart Arrest"[majr]** OR "Dysrhythmia"[ti] OR "Dysrhythmias"[ti] OR "Arrhythmia"[ti] OR "Arrhythmias"[ti] OR "Arrythmia"[ti] OR "Arrythmias"[ti] OR "Sick Sinus Syndrome"[ti] OR "Cardiac Sinus Arrest"[ti] OR "Atrial Fibrillation"[ti] OR "Atrial Flutter"[ti] OR "Bradycardia"[ti] OR "Brugada Syndrome"[ti] OR "Premature Cardiac Complexes"[ti] OR "Atrial Premature Complexes"[ti] OR "Ventricular Premature Complexes"[ti] OR "Commotio Cordis"[ti] OR "Heart Block"[ti] OR "Adams-Stokes Syndrome"[ti] OR "Atrioventricular Block"[ti] OR "Bundle-Branch Block"[ti] OR "Sinoatrial Block"[ti] OR "Long QT Syndrome"[ti] OR "Andersen Syndrome"[ti] OR "Jervell-Lange Nielsen Syndrome"[ti] OR "Romano-Ward Syndrome"[ti] OR "Parasystole"[ti] OR "Pre-Excitation Syndromes"[ti] OR "Lown-Ganong-Levine Syndrome"[ti] OR "Mahaim-type Pre-Excitation"[ti] OR "Wolff-Parkinson-White Syndrome"[ti] OR "Tachycardia"[ti] OR "Tachycardias"[ti] OR "Ventricular Fibrillation"[ti] OR "Ventricular Flutter"[ti] OR "heart rate variability"[ti] OR "Sudden Cardiac Death"[ti] OR "Sudden Cardiac Arrest"[ti] OR "asystole"[all fields] OR asystol*[all fields] **OR "heart arrest"[ti] OR "cardiac arrest"[ti])** AND ("Epilepsy"[majr] OR "epilepsy"[ti] OR "epileptic"[ti] OR "Epilepsies"[ti] OR "Landau-Kleffner Syndrome"[ti] OR "Epilepticus"[ti] OR "Epilepsia"[ti] OR epileps*[ti] OR epilept*[ti] OR "sudep"[all fields] OR sudden unexpected death in epilepsy OR sudden unexplained death in epilepsy)

("Arrhythmias, Cardiac"[mesh] OR "Dysrhythmia"[ti] OR "Dysrhythmias"[ti] OR "Arrhythmia"[ti] OR "Arrhythmias"[ti] OR "Arrythmia"[ti] OR "Arrythmias"[ti] OR "Sick Sinus Syndrome"[ti] OR "Cardiac Sinus Arrest"[ti] OR "Atrial Fibrillation"[ti] OR "Atrial Flutter"[ti] OR "Bradycardia"[ti] OR "Brugada Syndrome"[ti] OR "Premature Cardiac Complexes"[ti] OR "Atrial Premature Complexes"[ti] OR "Ventricular Premature Complexes"[ti] OR "Commotio Cordis"[ti] OR "Heart Block"[ti] OR "Adams-Stokes Syndrome"[ti] OR "Atrioventricular Block"[ti] OR "Bundle-Branch Block"[ti] OR "Sinoatrial Block"[ti] OR "Long QT Syndrome"[ti]

OR "Andersen Syndrome"[ti] OR "Jervell-Lange Nielsen Syndrome"[ti] OR "Romano-Ward Syndrome"[ti] OR "Parasystole"[ti] OR "Pre-Excitation Syndromes"[ti] OR "Lown-Ganong-Levine Syndrome"[ti] OR "Mahaim-type Pre-Excitation"[ti] OR "Wolff-Parkinson-White Syndrome"[ti] OR "Tachycardia"[ti] OR "Tachycardias"[ti] OR "Ventricular Fibrillation"[ti] OR "Ventricular Flutter"[ti] OR "heart rate variability"[ti] OR "asystole"[all fields] OR asystol*[all fields]) AND ("Epilepsy"[mesh] OR "epilepsy"[ti] OR "epileptic"[ti] OR "Epilepsies"[ti] OR "Landau-Kleffner Syndrome"[ti] OR "Epilepticus"[ti] OR "Epilepsia"[ti] OR epileps*[ti] OR epilept*[ti] OR "sudep"[all fields])

EMBASE
http://ovidsp.ovid.com/ovidweb.cgi?T=JS&PAGE=main&MODE=ovid&D=oemezd

(exp *heart arrhythmia / OR exp *Heart arrest/ OR "Dysrhythmia".ti OR "Dysrhythmias".ti OR "Arrhythmia".ti OR "Arrhythmias".ti OR "Arrythmia".ti OR "Arrythmias".ti OR "Sick Sinus Syndrome".ti OR "Cardiac Sinus Arrest".ti OR "Atrial Fibrillation".ti OR "Atrial Flutter".ti OR "Bradycardia".ti OR "Brugada Syndrome".ti OR "Premature Cardiac Complexes".ti OR "Atrial Premature Complexes".ti OR "Ventricular Premature Complexes".ti OR "Commotio Cordis".ti OR "Heart Block".ti OR "Adams-Stokes Syndrome".ti OR "Atrioventricular Block".ti OR "Bundle-Branch Block".ti OR "Sinoatrial Block".ti OR "Long QT Syndrome".ti OR "Andersen Syndrome".ti OR "Jervell-Lange Nielsen Syndrome".ti OR "Romano-Ward Syndrome".ti OR "Parasystole".ti OR "Pre-Excitation Syndromes".ti OR "Lown-Ganong-Levine Syndrome".ti OR "Mahaim-type Pre-Excitation".ti OR "Wolff-Parkinson-White Syndrome".ti OR "Tachycardia".ti OR "Tachycardias".ti OR "Ventricular Fibrillation".ti OR "Ventricular Flutter".ti OR "heart rate variability".ti OR "Sudden Cardiac Death".ti OR "Sudden Cardiac Arrest".ti OR "asystole".mp OR asystol*.mp OR "heart arrest".ti OR "cardiac arrest".ti) AND (exp *Epilepsy/ OR "epilepsy".ti OR "epileptic".ti OR "Epilepsies".ti OR "Landau-Kleffner Syndrome".ti OR "Epilepticus".ti OR "Epilepsia".ti OR epileps*.ti OR epilept*.ti OR "sudep".mp OR sudden unexpected death in epilepsy.mp OR sudden unexplained death in epilepsy.mp)

Web of Science
http://isiknowledge.com/wos

(TI=((heart arrhythmia OR Heart arrest OR Dysrhythmia OR Dysrhythmias OR Arrhythmia OR Arrhythmias OR Arrythmia OR Arrythmias OR Sick Sinus Syndrome OR Cardiac Sinus Arrest OR Atrial Fibrillation OR Atrial Flutter OR Bradycardia OR Brugada Syndrome OR Premature Cardiac Complexes OR Atrial Premature Complexes OR Ventricular Premature Complexes OR Commotio Cordis OR Heart Block OR Adams-Stokes Syndrome OR Atrioventricular Block OR Bundle-Branch Block OR Sinoatrial Block OR Long QT

Syndrome OR Andersen Syndrome OR Jervell-Lange Nielsen Syndrome OR Romano-Ward Syndrome OR Parasystole OR Pre-Excitation Syndromes OR Lown-Ganong-Levine Syndrome OR Mahaim-type Pre-Excitation OR Wolff-Parkinson-White Syndrome OR Tachycardia OR Tachycardias OR Ventricular Fibrillation OR Ventricular Flutter OR heart rate variability OR Sudden Cardiac Death OR Sudden Cardiac Arrest OR asystole OR asystol* OR cardiac arrest) AND (Epilepsy OR epilepsy OR epileptic OR Epilepsies OR Landau-Kleffner Syndrome OR Epilepticus OR Epilepsia OR epileps* OR epilept* OR sudep OR sudden unexpected death in epilepsy OR sudden unexplained death in epilepsy))) OR (TI=("heart arrhythmia" OR "Heart arrest" OR "Dysrhythmia" OR "Dysrhythmias" OR "Arrhythmia" OR "Arrhythmias" OR "Arrythmia" OR "Arrythmias" OR "Sick Sinus Syndrome" OR "Cardiac Sinus Arrest" OR "Atrial Fibrillation" OR "Atrial Flutter" OR "Bradycardia" OR "Brugada Syndrome" OR "Premature Cardiac Complexes" OR "Atrial Premature Complexes" OR "Ventricular Premature Complexes" OR "Commotio Cordis" OR "Heart Block" OR "Adams-Stokes Syndrome" OR "Atrioventricular Block" OR "Bundle-Branch Block" OR "Sinoatrial Block" OR "Long QT Syndrome" OR "Andersen Syndrome" OR "Jervell-Lange Nielsen Syndrome" OR "Romano-Ward Syndrome" OR "Parasystole" OR "Pre-Excitation Syndromes" OR "Lown-Ganong-Levine Syndrome" OR "Mahaim-type Pre-Excitation" OR "Wolff-Parkinson-White Syndrome" OR "Tachycardia" OR "Tachycardias" OR "Ventricular Fibrillation" OR "Ventricular Flutter" OR "heart rate variability" OR "Sudden Cardiac Death" OR "Sudden Cardiac Arrest" OR "asystole" OR asystol* OR "cardiac arrest") AND TS=("sudep" OR "sudden unexpected death in epilepsy" OR "sudden unexplained death in epilepsy"))

Cochrane
http://www.thecochranelibrary.com/view/0/index.html

(heart arrhythmia OR Heart arrest OR Dysrhythmia OR Dysrhythmias OR Arrhythmia OR Arrhythmias OR Arrythmia OR Arrythmias OR Sick Sinus Syndrome OR Cardiac Sinus Arrest OR Atrial Fibrillation OR Atrial Flutter OR Bradycardia OR Brugada Syndrome OR Premature Cardiac Complexes OR Atrial Premature Complexes OR Ventricular Premature Complexes OR Commotio Cordis OR Heart Block OR Adams-Stokes Syndrome OR Atrioventricular Block OR Bundle-Branch Block OR Sinoatrial Block OR Long QT Syndrome OR Andersen Syndrome OR Jervell-Lange Nielsen Syndrome OR Romano-Ward Syndrome OR Parasystole OR Pre-Excitation Syndromes OR Lown-Ganong-Levine Syndrome OR Mahaim-type Pre-Excitation OR Wolff-Parkinson-White Syndrome OR Tachycardia OR Tachycardias OR Ventricular Fibrillation OR Ventricular Flutter OR heart rate variability OR Sudden Cardiac Death OR Sudden Cardiac Arrest OR asystole OR asystol* OR cardiac arrest) AND (Epilepsy OR epilepsy OR epileptic OR Epilepsies OR Landau-Kleffner Syndrome OR Epilepticus OR Epilepsia OR epileps* OR epilept* OR sudep OR sudden unexpected death in epilepsy OR sudden unexplained death in epilepsy)
