# Supplementary material for: Cardiac arrhythmias during or after epileptic seizures
Source: J Neurol Neurosurg Psychiatry. 2015 Jun 2;87(1):69–74. doi: 10.1136/jnnp-2015-310559 (PMC4717443; doi:10.1136/jnnp-2015-310559)
Supplement: Web appendix 2 [file jnnp-2015-310559-s2.pdf]

Appendix B - References per arrhythmia

Ictal asystole

1. Agostini SD, Aniles E, Sirven J, et al. The importance of cardiac monitoring in the epilepsy monitoring unit: a case presentation of ictal asystole. NeurodiagnJ 2012;**52**(3):250-60
2. Bae EK, Park K, Kim H, et al. Ictal asystole and eating reflex seizures with temporal lobe epilepsy. Epilepsy Behav 2011;**20**(2):404-06 doi: S1525-5050(10)00755-9 [pii];10.1016/j.yebeh.2010.12.010 [doi][published Online First: Epub Date]|.
3. Beal JC, Sogawa Y, Ceresnak SR, et al. Late onset ictal asystole in refractory epilepsy. Pediatr Neurol 2011;**45**(4):253-55 doi: S0887-8994(11)00318-3 [pii];10.1016/j.pediatrneurol.2011.07.005 [doi][published Online First: Epub Date]|.
4. Britton JW, Ghearing GR, Benarroch EE, et al. The ictal bradycardia syndrome: localization and lateralization. Epilepsia 2006;**47**(4):737-44 doi: EPI509 [pii];10.1111/j.1528-1167.2006.00509.x [doi][published Online First: Epub Date]|.
5. Carvalho KS, Salanova V, Markand ON. Cardiac asystole during a temporal lobe seizure. Seizure 2004;**13**(8):595-99 doi: S1059131104000159 [pii];10.1016/j.seizure.2004.01.004 [doi][published Online First: Epub Date]|.
6. Devinsky O, Pacia S, Tatambhotla G. Bradycardia and asystole induced by partial seizures: a case report and literature review. Neurology 1997;**48**(6):1712-14
7. Duplyakov D GG, Lyukshina N, Surkova E, Elger CE, Surges R. Syncope, seizure-induced bradycardia and asystole: Two cases and review of clinical and pathofysiological features. Seizure 2014
8. Fincham RW, Shivapour ET, Leis AA, et al. Ictal bradycardia with syncope: a case report. Neurology 1992;**42**(11):2222-23
9. Gemma LW, Olson JA, Padanilam BJ, et al. Complex-partial seizure causing prolonged asystole. Journal of Cardiovascular Electrophysiology 2013;**24**(3):356-57
10. Kang DY, Oh IY, Lee SR, et al. Recurrent syncope triggered by temporal lobe epilepsy: ictal bradycardia syndrome. Korean CircJ 2012;**42**(5):349-51 doi: 10.4070/kcj.2012.42.5.349 [doi][published Online First: Epub Date]|.
11. Kerling F, Dutsch M, Linke R, et al. Relation between ictal asystole and cardiac sympathetic dysfunction shown by MIBG-SPECT. Acta Neurol Scand 2009;**120**(2):123-29 doi: ANE1135 [pii];10.1111/j.1600-0404.2008.01135.x [doi][published Online First: Epub Date]|.
12. Lanz M, Oehl B, Brandt A, et al. Seizure induced cardiac asystole in epilepsy patients undergoing long term video-EEG monitoring. Seizure 2011;**20**(2):167-72 doi: S1059-1311(10)00300-6 [pii];10.1016/j.seizure.2010.11.017 [doi][published Online First: Epub Date]|.
13. Liedholm LJ, Gudjonsson O. Cardiac arrest due to partial epileptic seizures. Neurology 1992;**42**(4):824-29
14. Lim EC, Lim SH, Wilder-Smith E. Brain seizes, heart ceases: a case of ictal asystole. J Neurol NeurosurgPsychiatry 2000;**69**(4):557-59
15. Locatelli ER, Varghese JP, Shuaib A, et al. Cardiac asystole and bradycardia as a manifestation of left temporal lobe complex partial seizure. AnnInternMed

1999;**130**(7):581-83 doi: 199904060-00007 [pii][published Online First: Epub Date]|.
16. Marynissen T, Govers N, Vydt T. Ictal asystole: case report with review of literature. Acta Cardiol 2012;**67**(4):461-64
17. Mascia A, Quarato PP, Sparano A, et al. Cardiac asystole during right frontal lobe seizures: a case report. Neurol Sci 2005;**26**(5):340-43 doi: 10.1007/s10072-005-0496-4 [doi][published Online First: Epub Date]|.
18. Nguyen-Michel VH, Adam C, Dinkelacker V, et al. Characterization of seizure-induced syncopes: EEG, ECG, and clinical features. Epilepsia 2014;**55**(1):146-55 doi: 10.1111/epi.12482[published Online First: Epub Date]|.
19. Panchani J, Adjei P, Henneberger C, et al. Asymmetric hemispheric representation of periictal heart rate modulation is individually lateralised. Epileptic Disord 2011;**13**(2):172-76 doi: epd.2011.0430 [pii];10.1684/epd.2011.0430 [doi][published Online First: Epub Date]|.
20. Reeves AL, Nollet KE, Klass DW, et al. The ictal bradycardia syndrome. Epilepsia 1996;**37**(10):983-87
21. Rocamora R, Kurthen M, Lickfett L, et al. Cardiac asystole in epilepsy: clinical and neurophysiologic features. Epilepsia 2003;**44**(2):179-85 doi: epi15101 [pii][published Online First: Epub Date]|.
22. Rossetti AO, Dworetzky BA, Madsen JR, et al. Ictal asystole with convulsive syncope mimicking secondary generalisation: a depth electrode study. J Neurol NeurosurgPsychiatry 2005;**76**(6):885-87 doi: 76/6/885 [pii];10.1136/jnnp.2004.051839 [doi][published Online First: Epub Date]|.
23. Rubboli G, Bisulli F, Michelucci R, et al. Sudden falls due to seizure-induced cardiac asystole in drug-resistant focal epilepsy. Neurology 2008;**70**(20):1933-35 doi: 01.wnl.0000289193.89796.83 [pii];10.1212/01.wnl.0000289193.89796.83 [doi][published Online First: Epub Date]|.
24. Rugg-Gunn FJ, Duncan JS, Smith SJ. Epileptic cardiac asystole. J Neurol NeurosurgPsychiatry 2000;**68**(1):108-10
25. Schuele SU, Bermeo AC, Alexopoulos AV, et al. Video-electrographic and clinical features in patients with ictal asystole. Neurology 2007;**69**(5):434-41 doi: 69/5/434 [pii];10.1212/01.wnl.0000266595.77885.7f [doi][published Online First: Epub Date]|.
26. Scott CA, Fish DR. Cardiac asystole in partial seizures. Epileptic Disord 2000;**2**(2):89-92
27. Serafini A, Gelisse P, Reana V, et al. Cardiac asystole during a cluster of right temporo-parietal seizures. Seizure 2011;**20**(2):181-83 doi: S1059-1311(10)00262-1 [pii];10.1016/j.seizure.2010.10.031 [doi][published Online First: Epub Date]|.
28. Steckman D, Katz D, Sauer W, et al. Seizure-induced asystole. Heart 2011;**97**(17):1457 doi: heartjnl-2011-300277 [pii];10.1136/heartjnl-2011-300277 [doi][published Online First: Epub Date]|.
29. Winesett P, Feliciano CA, Tatum WO. Temporal lobe seizures triggering recurrent syncope by ictal asystole. Epilepsy Behav 2009;**14**(1):258-60 doi: S1525-5050(08)00286-2 [pii];10.1016/j.yebeh.2008.09.020 [doi][published Online First: Epub Date]|.
30. Wittekind SG, Lie O, Hubbard S, et al. Ictal asystole: an indication for pacemaker implantation and emerging cause of sudden death. Pacing Clin Electrophysiol

2012;**35**(7):e193-e96 doi: 10.1111/j.1540-8159.2011.03179.x [doi][published Online First: Epub Date]|.
31. Wolber T, Namdar M, Duru F. Heart obeys the brain: seizure ceases cardiac rhythm. Pacing Clin Electrophysiol 2010;**33**(8):e72-e75 doi: PACE2712 [pii];10.1111/j.1540-8159.2010.02712.x [doi][published Online First: Epub Date]|.
32. Zubair S, Arshad AB, Saeed B, et al. Ictal asystole--late manifestation of partial epilepsy and importance of cardiac pacemaker. Seizure 2009;**18**(6):457-61 doi: S1059-1311(09)00050-8 [pii];10.1016/j.seizure.2009.03.004 [doi][published Online First: Epub Date]|.
33. Kouakam C, Daems C, Guedon-Moreau L, et al. Recurrent unexplained syncope may have a cerebral origin: report of 10 cases of arrhythmogenic epilepsy. Arch CardiovascDis 2009;**102**(5):397-407 doi: S1875-2136(09)00085-0 [pii];10.1016/j.acvd.2009.02.014 [doi][published Online First: Epub Date]|.
34. Chaila E, Bhangu J, Tirupathi S, et al. Ictal bradycardia and asystole associated with intractable epilepsy: A case series. British Journal of Cardiology 2010;**17**(5):245-48
35. Odier C, Nguyen DK, Bouthillier A, et al. Potentially life-threatening ictal bradycardia in intractable epilepsy. CanJ Neurol Sci 2009;**36**(1):32-35
36. Strzelczyk A, Cenusa M, Bauer S, et al. Management and long-term outcome in patients presenting with ictal asystole or bradycardia. Epilepsia 2011;**52**(6):1160-7 doi: 10.1111/j.1528-1167.2010.02961.x[published Online First: Epub Date]|.

<u>Postictal aystole</u>

1. Lanz M, Oehl B, Brandt A, et al. Seizure induced cardiac asystole in epilepsy patients undergoing long term video-EEG monitoring. Seizure 2011;**20**(2):167-72 doi: S1059-1311(10)00300-6 [pii];10.1016/j.seizure.2010.11.017 [doi][published Online First: Epub Date]|.
2. Rocamora R, Kurthen M, Lickfett L, et al. Cardiac asystole in epilepsy: clinical and neurophysiologic features. Epilepsia 2003;**44**(2):179-85 doi: epi15101 [pii][published Online First: Epub Date]|.
3. Seeck M, Blanke O, Jallon P, et al. Symptomatic postictal cardiac asystole in a young patient with partial seizures. Europace 2001;**3**(3):247-52 doi: S1099-5129(01)90173-4 [pii];10.1053/eupc.2001.0173 [doi][published Online First: Epub Date]|.
4. Mehvari JF, F. Cardiac arrest associated with epileptic seizures: A case report with simultaneous EEG and ECG. Epilepsy & Behavior Case Reports 2014(2):145-51
5. Ryvlin P. Incidence and mechanisms of cardiorespiratory arrests in epilepsy monitoring units (MORTEMUS): a retrospective study. Lancet Neurology 2013

<u>Ictal bradycardia</u>

1. Almansori M, Ijaz M, Ahmed SN. Cerebral arrhythmia influencing cardiac rhythm: a case of ictal bradycardia. Seizure 2006;**15**(6):459-61 doi: S1059-1311(06)00114-2 [pii];10.1016/j.seizure.2006.05.008 [doi][published Online First: Epub Date]|.

2. Blumhardt LD, Smith PEM, Owen L. Electrocardiographic accompaniments of temporal lobe epileptic seizures. Lancet 1986;**1**(8489):1051-56
3. Jacome DE, Seroppian ER. Ictal bradycardia. Am J Med Sci 1988;**295**(5):469-71
4. Kahane P, Di LM, Hoffmann D, et al. Ictal bradycardia in a patient with a hypothalamic hamartoma: a stereo-EEG study. Epilepsia 1999;**40**(4):522-27
5. van RK, Saussu F, de BT. Bradycardia, an epileptic ictal manifestation. Seizure 1995;**4**(3):237-39
6. Wilder-Smith E. Complete atrio-ventricular conduction block during complex partial seizure. J Neurol NeurosurgPsychiatry 1992;**55**(8):734-36
7. Britton JW, Ghearing GR, Benarroch EE, et al. The ictal bradycardia syndrome: localization and lateralization. Epilepsia 2006;**47**(4):737-44 doi: EPI509 [pii];10.1111/j.1528-1167.2006.00509.x [doi][published Online First: Epub Date]|.
8. Reeves AL, Nollet KE, Klass DW, et al. The ictal bradycardia syndrome. Epilepsia 1996;**37**(10):983-87
9. Tinuper P, Bisulli F, Cerullo A, et al. Ictal bradycardia in partial epileptic seizures: Autonomic investigation in three cases and literature review. Brain 2001;**124**(Pt 12):2361-71
10. Kouakam C, Daems-Monpeurt C, Le FP, et al. [Complete atrioventricular block during temporal lobe epilepsy. Apropos of a case]. Arch Mal Coeur Vaiss 1999;**92**(2):265-68
11. Odier C, Nguyen DK, Bouthillier A, et al. Potentially life-threatening ictal bradycardia in intractable epilepsy. CanJ Neurol Sci 2009;**36**(1):32-35
12. Strzelczyk A, Cenusa M, Bauer S, et al. Management and long-term outcome in patients presenting with ictal asystole or bradycardia. Epilepsia 2011;**52**(6):1160-7 doi: 10.1111/j.1528-1167.2010.02961.x[published Online First: Epub Date]|.

<u>(Post)Ictal AV-block</u>

1. Altenmuller DM, Zehender M, Schulze-Bonhage A. High-grade atrioventricular block triggered by spontaneous and stimulation-induced epileptic activity in the left temporal lobe. Epilepsia 2004;**45**(12):1640-44 doi: EPI34403 [pii];10.1111/j.0013-9580.2004.34403.x [doi][published Online First: Epub Date]|.
2. Kouakam C, Daems-Monpeurt C, Le FP, et al. [Complete atrioventricular block during temporal lobe epilepsy. Apropos of a case]. Arch Mal Coeur Vaiss 1999;**92**(2):265-68
3. Kouakam C, Daems C, Guedon-Moreau L, et al. Recurrent unexplained syncope may have a cerebral origin: report of 10 cases of arrhythmogenic epilepsy. Arch CardiovascDis 2009;**102**(5):397-407 doi: S1875-2136(09)00085-0 [pii];10.1016/j.acvd.2009.02.014 [doi][published Online First: Epub Date]|.
4. Lanz M, Oehl B, Brandt A, et al. Seizure induced cardiac asystole in epilepsy patients undergoing long term video-EEG monitoring. Seizure 2011;**20**(2):167-72 doi: S1059-1311(10)00300-6 [pii];10.1016/j.seizure.2010.11.017 [doi][published Online First: Epub Date]|.
5. Opherk C, Coromilas J, Hirsch LJ. Heart rate and EKG changes in 102 seizures: analysis of influencing factors. Epilepsy Res 2002;**52**(2):117-27

6. Surges R, Scott CA, Walker MC. Peri-ictal atrioventricular conduction block in a patient with a lesion in the left insula: case report and review of the literature. Epilepsy Behav 2009;**16**(2):347-49 doi: S1525-5050(09)00426-0 [pii];10.1016/j.yebeh.2009.07.036 [doi][published Online First: Epub Date]|.
7. Wilder-Smith E. Complete atrio-ventricular conduction block during complex partial seizure. J Neurol NeurosurgPsychiatry 1992;**55**(8):734-36
8. Gordon S, Saksena S, Parsonnet V. Latent intraHisian block provoked by a seizure. Am Heart J 1987;**113**(3):837-39
9. Tigaran S, Molgaard H, Dam M. Atrio-ventricular block: a possible explanation of sudden unexpected death in epilepsy. Acta Neurol Scand 2002;**106**(4):229-33 doi: 2c017 [pii][published Online First: Epub Date]|.

Atrial fibrillation

1. Nei M, Ho RT, Sperling MR. EKG abnormalities during partial seizures in refractory epilepsy. Epilepsia 2000;**41**(5):542-48
2. Nei M. EEG and ECG in Sudden Unexplained Death in Epilepsy. Epilepsia 2004;**45**(4):338-45
3. Herskovitz M, Schiller Y. Atrial fibrillation associated with epileptic seizures. Arch Neurol 2012;**69**(9):1197-99 doi: 1169806 [pii];10.1001/archneurol.2011.3647 [doi][published Online First: Epub Date]|.
4. Mathew NT, Taori GM, Mathai KV, et al. Atrial fibrillation associated with seizure in a case of frontal meningioma. Neurology 1970;**20**(7):725-28
5. Vedovello M, Baldacci F, Nuti A, et al. Peri-ictal prolonged atrial fibrillation after generalized seizures: description of a case and etiopathological considerations. Epilepsy Behav 2012;**23**(3):377-78 doi: S1525-5050(12)00020-0 [pii];10.1016/j.yebeh.2012.01.005 [doi][published Online First: Epub Date]|.
6. Tigaran S DM. Atrial fibrillation: an overlooked complication of epileptic seizures? Epilepsia 1998
7. Surges R, Moskau S, Viebahn B, et al. Prolonged atrial fibrillation following generalized tonic-clonic seizures. Seizure 2012;**21**(8):643-45 doi: S1059-1311(12)00141-0 [pii];10.1016/j.seizure.2012.05.012 [doi][published Online First: Epub Date]|.

Ventricular fibrillation

1. Espinosa PS, Lee JW, Tedrow UB, et al. Sudden unexpected near death in epilepsy: malignant arrhythmia from a partial seizure. Neurology 2009;**72**(19):1702-03 doi: 72/19/1702 [pii];10.1212/WNL.0b013e3181a55f90 [doi][published Online First: Epub Date]|.
2. Ferlisi M, Tomei R, Carletti M, et al. Seizure induced ventricular fibrillation: a case of near-SUDEP. Seizure 2013;**22**(3):249-51 doi: S1059-1311(12)00329-9 [pii];10.1016/j.seizure.2012.12.008 [doi][published Online First: Epub Date]|.
3. Jeppesen J, Fuglsang-Frederiksen A, Brugada R, et al. Heart rate variability analysis indicates preictal parasympathetic overdrive preceding seizure-induced cardiac dysrhythmias leading to sudden unexpected death in a patient with epilepsy. Epilepsia 2014 doi: 10.1111/epi.12614[published Online First: Epub Date]|.
